# A Short Case Report on Ruptured Sinus of Valsalva Aneurysm

**DOI:** 10.7759/cureus.10263

**Published:** 2020-09-05

**Authors:** Abhishek Matta, Arun K Nagabandi, Dinesh Bande

**Affiliations:** 1 Internal Medicine, University of North Dakota School of Medicine, Fargo, USA; 2 Interventional Cardiology, Pikeville Medical Center, Pikeville, USA

**Keywords:** ruptured sinus of valsalva aneurysm, aorto-atrial fistula, aorto-right atrial fistula, percutaneous closure of ruptured sinus of valsalva aneurysm, sinus of valsalva aneurysm, left to right shunt

## Abstract

A 35-year-old woman without any history of congenital heart disease presented to our clinic with dyspnea on exertion. Transthoracic echocardiogram (TTE) showed an eccentric tricuspid regurgitant jet and increased right ventricular systolic pressure. Transesophageal echocardiogram (TEE) revealed a sinus of Valsalva aneurysm (SVA) arising from the noncoronary sinus that ruptured into the right atrium, leading to the formation of an aorto-right atrial fistula. Right heart catheterization confirmed left to right shunt. The fistulous tract was resected, and the aneurysm repaired surgically. The patient made a good recovery.

## Introduction

Sinus of Valsalva aneurysm (SVA) is defined as an abnormal dilatation or enlargement of one or more of the aortic sinuses between the aortic valve annulus and the sinotubular junction. SVAs can be congenital or acquired. SVAs are found in 0.09% of the general population [1] and 0.15%-1.5% of patients undergoing heart surgery [2]. Typically men are more affected than women (4:1), and higher incidence is reported among Asians [3]. A majority of the SVAs are congenital but can be acquired secondary to infective endocarditis, trauma, systemic inflammatory diseases, connective tissue diseases like Marfan syndrome, or atherosclerosis [2]. SVAs account for 0.1%-3.5% of all congenital cardiac defects [3]. SVAs can rupture and form fistulous tracts from the aorta into the adjoining chambers of the heart, which can lead to congestive heart failure [1]. Congenital SVAs classically rupture between 20 and 40 years of age [3]. This report describes the case of a patient who presented with dyspnea on exertion and was diagnosed with a ruptured SVA aneurysm into the right atrium, leading to an aorto-right atrial fistula. This case report highlights the importance of thorough cardiac workup in young patients who present with unexplained dyspnea without any major comorbidities.

## Case presentation

**Figure 1:**
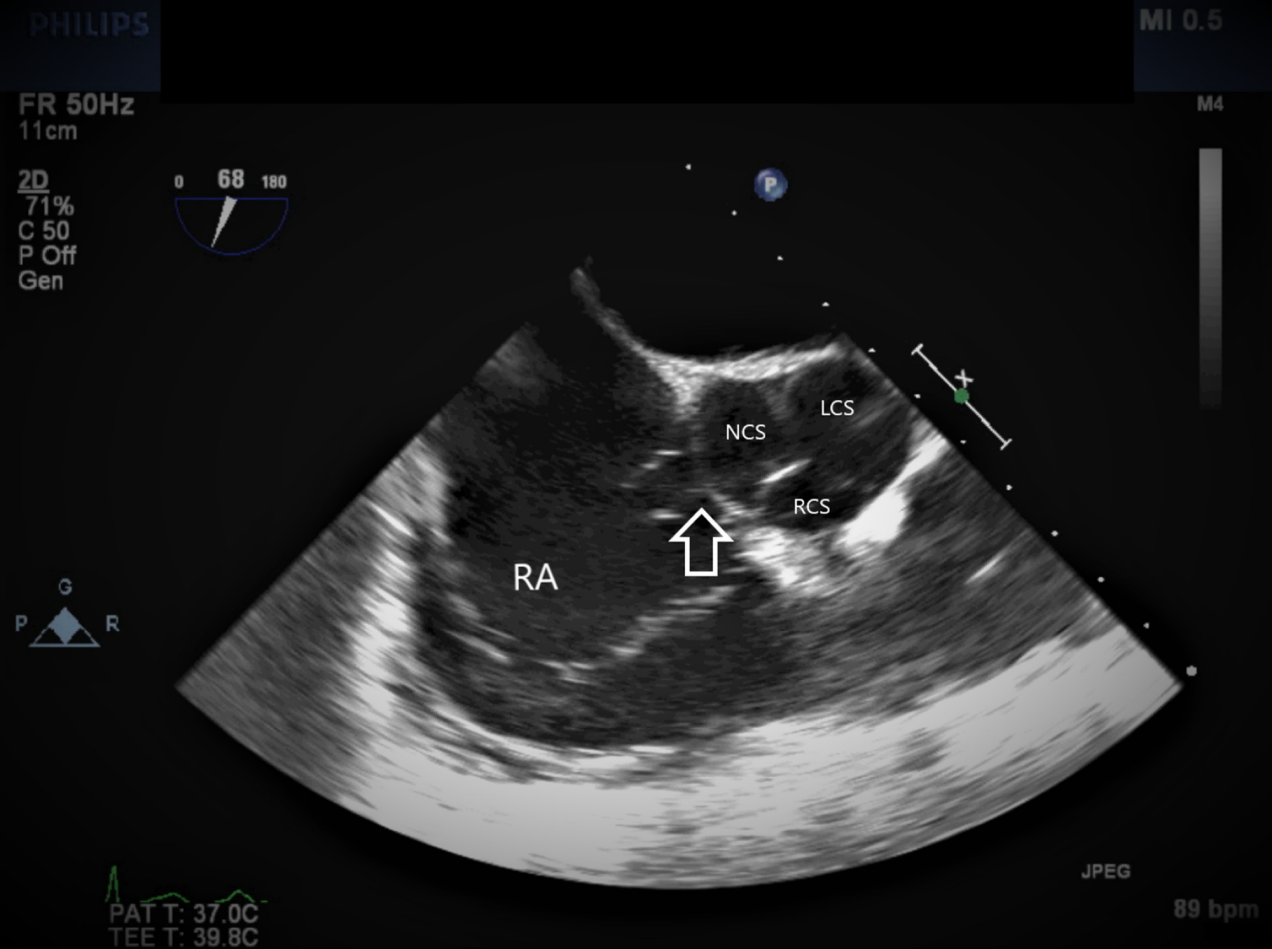
TEE image at mid-esophageal aortic valve short axis view The arrow showing SVA arising from the noncoronary sinus. TEE, transesophageal echocardiogram; SVA, sinus of Valsalva aneurysm; RA, right atrium; NCS, noncoronary sinus; LCS, left coronary sinus; RCS, right coronary sinus.

**Figure 2:**
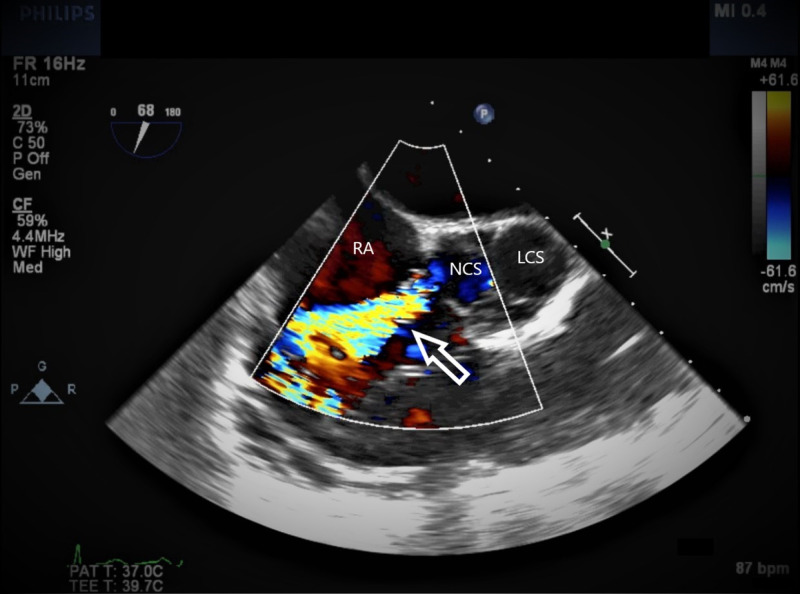
TEE with color Doppler The arrow indicating the high-velocity jet from the NCS into the RA through the ruptured SVA. TEE, transesophageal echocardiogram; SVA, sinus of Valsalva aneurysm; RA, right atrium; NCS, noncoronary sinus; LCS, left coronary sinus.

A 35-year-old African-American woman presented to the cardiology clinic with dyspnea on exertion for six months. She denied any chest pain or palpitations. She did not report any history of congenital heart disease, tobacco or alcohol use, or any family history of cardiac problems. Vitals were heart rate 85 beats/min, blood pressure 110/70 mm Hg, temperature 36.9˚C, and body mass index 25. Physical examination revealed a normal S1, loud S2 in the pulmonary area, S4, and a grade 3 systolic murmur in the pulmonary area. Electrocardiogram (EKG) showed normal sinus rhythm and an incomplete right bundle branch block. Based on symptoms and physical examination, our differential diagnosis included anemia, congestive heart failure, and lung disease. Hemoglobin was 11.8 g/dL (normal 11.5-15.2 g/dL). The chest X-ray showed clear lung fields. Transthoracic echocardiogram (TTE) showed a dilated right atrium, a dilated right ventricle, and an eccentric tricuspid regurgitant jet, and the right ventricular systolic pressure (RVSP) was estimated to be 76 mm Hg. Transmitral spectral flow Doppler showed a restrictive pattern. Transesophageal echocardiogram (TEE) clearly delineated an aneurysm of the sinus of Valsalva arising from the noncoronary sinus that ruptured into the right atrium (Figures 1, 2), creating an aorto-right atrial fistula. Right heart catheterization showed a step-up of oxygen saturations from 63% in the superior venacava to 75% in the right atrium and 81% in the right ventricle confirming the aorto-right atrial fistula with left to right shunt. Right atrial pressure was 8 mm Hg, right ventricular pressure was 41/4 mm Hg, pulmonary artery pressure was 38/17 mm Hg, and pulmonary wedge pressure was 15 mm Hg.

**Figure 3:**
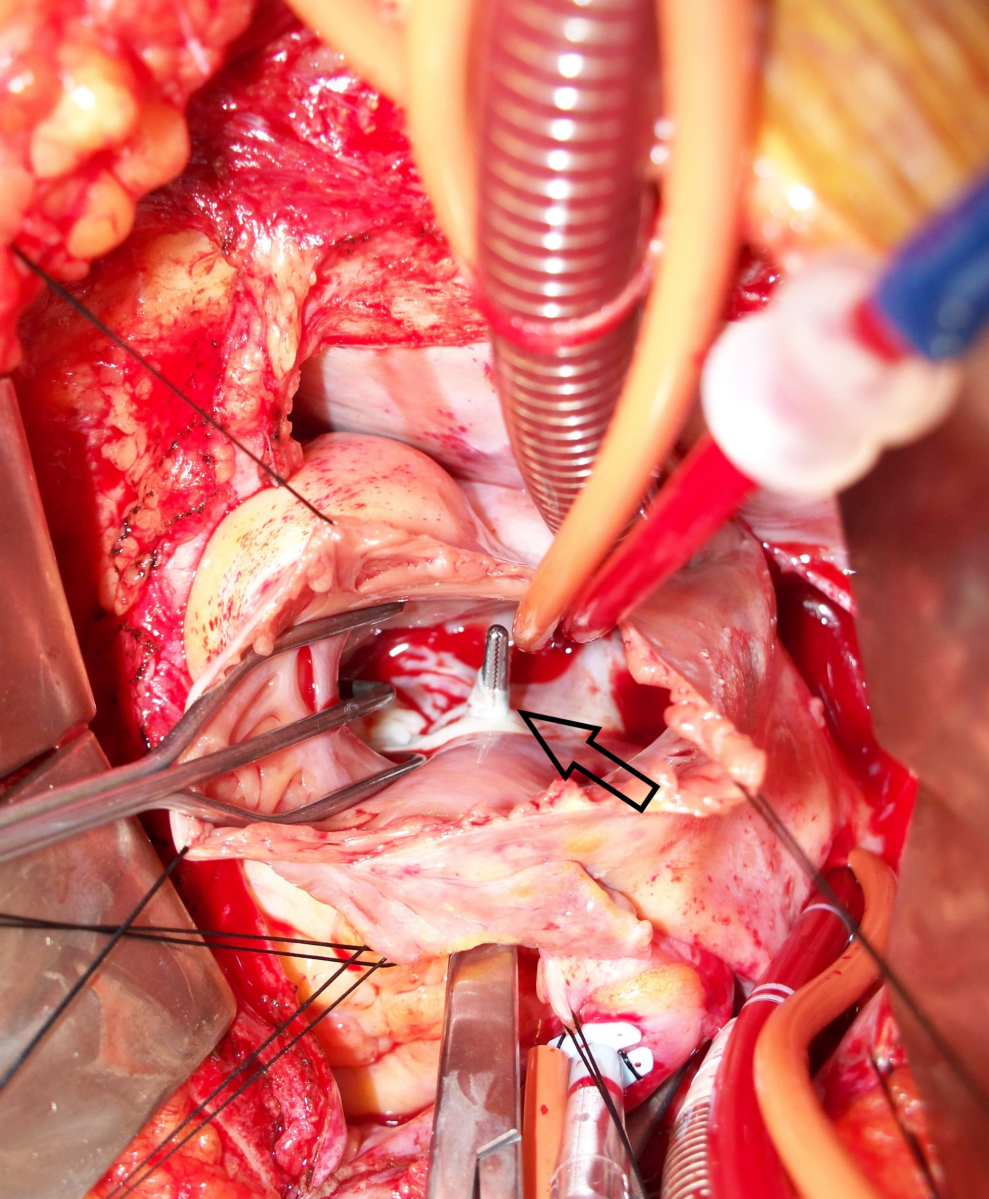
Intraoperative image Hemostat (indicated by arrow) showing the defect between right atrium and noncoronary sinus.

The investigations were completed within two weeks of her initial presentation to the clinic, and we decided to perform an elective sinus of Valsalva repair to improve her survival. The ruptured sinus of Valsalva along with the fistulous tract was easily identified during the procedure (Figure 3). It was at the level of the central fibrous body on the atrial side of the tricuspid valve. The fistulous tract also involved the base of the aortic valve on the aortic side. The fistulous tract was resected, and the aortic defect was closed involving a portion of the base of the aortic valve. The patient tolerated the procedure well. She remained in the hospital for a total of five days and made a good recovery.

## Discussion

SVA is the result of the weakness of the elastic lamina at the junction of the aortic media and the annulus fibrosis [1]. Aneurysms of the right and noncoronary sinuses are more common than the left sinus [4]. Rupture with fistulous tract formation is a potential complication of SVA, which can lead to shunting and cardiac failure [1]. Noncoronary sinus aneurysms tend to rupture into the right atrium, and right coronary sinus aneurysms can rupture into either the right atrium or right ventricle [5]. Ruptured SVA can present with dyspnea, palpitations, fatigue, syncope, chest pain, or decreased exercise tolerance [1,6]. TTE or TEE with Doppler is the first line of imaging although CT and cardiac MRI can provide an excellent anatomic depiction of either ruptured or unruptured aneurysm [6,7]. The median survival of untreated ruptured SVA is 3.9 years [8]. Ruptured SVAs are traditionally managed surgically with good outcomes [5,9]. The operative mortality rate is 1.9%-3.6% with 90% survival at 15 years [3]. The accurate mortality associated with percutaneous closure of ruptured SVA could not be determined due to paucity of large-scale data, but multiple studies have shown good outcomes in select patients [10-13]. The choice of percutaneous vs surgical intervention depends on multiple factors, including patient’s age, comorbidities, hemodynamic status at presentation, coexisting congenital heart defects like ventricular septal defects, aortic regurgitation, underlying obstructive coronary artery disease, size of ruptured SVA, and location in addition to the local cardiac surgeon and interventional cardiologist's expertise. The decision is best made by a multidisciplinary team. 

Our patient had a ruptured SVA arising from the noncoronary sinus with a fistulous tract into the right atrium. The aneurysm was probably congenital as she did not have any comorbidities and the age of presentation was classic for congenital SVA rupture. TTE only showed what appeared to be an eccentric tricuspid regurgitant jet, but TEE clearly showed the SVA and the fistulous tract. Our patient also had a left to right shunt as evidenced by the step-up in oxygen saturation from the superior venacava to the right atrium during right heart catheterization. We decided to manage the fistula surgically as our patient was young and hence, a good surgical candidate. The outcome was successful.

## Conclusions

SVA involving the noncoronary sinus can rupture into the right atrium and lead to an aorto-atrial fistula. The fistulous tract will cause left to right shunting and will lead to congestive heart failure if not managed in a timely fashion. Common symptoms include dyspnea, palpitations, fatigue, and decreased exercise tolerance even in a young patient without comorbidities. Noninvasive imaging like TTE, TEE, CT, and cardiac MRI can identify the aneurysm and fistulous tract. Ruptured SVAs are usually managed surgically. In select patients, percutaneous closure is a safe alternative to surgery, but long-term follow-up studies are necessary. 
